# Audit Cycle of Record Keeping by Doctors in Older Adult Inpatient Settings

**DOI:** 10.1192/bjo.2025.10549

**Published:** 2025-06-20

**Authors:** E Betty Anthony, Damola Akinmoladun, Dumogo Anochie, Oluyemi Akinmolayan

**Affiliations:** 1 Cumbria Northumberland Tyne and Wear NHS Foundation Trust, Newcastle, United Kingdom; 2 South Tees NHS Foundation Trust, Middlesbrough, United Kingdom; 3 Northamptonshire Healthcare NHS Foundation Trust, Northamptonshire, United Kingdom

## Abstract

**Aims:** To assess compliance with record keeping policies.Medical records play a vital role in supporting patient care. However, effective record keeping in clinical practice, particularly in mental health, poses significant challenges. The General Medical Council’s (GMC) good medical practice, states that doctors must ensure their records are clear, accurate, and legible. Regulation 28 of the Coroners and Justice Act 2009 empowers coroners to address concerns that could lead to future deaths. Davies Arnold Cooper (DAC) Beacroft’s 2022 report identified record keeping as a key issue in mental health.

**Methods:** Patients across three older adult inpatient wards were identified using convenience sampling method. Five hundred and thirty-three entries made by doctors for the audit and 424 entries made by doctors for the re-audit were identified using patient identifier. Data compilation was done using Excel spreadsheet and analysed using descriptive statistics. Outpatient entries and ECT entries made by doctors were excluded, ensuring a focused assessment of inpatient records. The results were presented using bar charts, pie charts and tables.

**Results:** The results were compared with the trust’s Record Management Policy and the previous audit conducted in January 2023. The re-audit found an improvement in the percentage of validated entries across all three wards compared with the previous audit. One of the wards showed the highest improvement, with a 35% increase in validated entries. However, the overall validation rate was still below the 80% requirement standard set. The timescales for validation across the three wards also showed some improvement, with the majority of validated entries meeting the 12-hour standard, although a small percentage remained unvalidated for longer periods. In addition, doctors were more likely to sign off their entries during normal work hours than out of hours

**Conclusion:** The findings suggest that while there has been improvement in the timeliness and completeness of clinical entries validation, more work is needed to ensure full compliance with the trust’s policies and the GMC’s good medical practice. Recommendations include regular reminders to doctors on promptly signing off clinical entries, incorporating record-keeping guidelines into local inductions, and a review of the trust’s guidelines on note validation for inpatient entries. This audit cycle led to a broader quality improvement project and trustwide policy change on validation of clinical entries. It highlights the importance of maintaining accurate and timely clinical entries.

